# Effect of Intrathecal Midazolam in the Severity of Pain in Cesarean Section: A Randomized Controlled Trail

**Published:** 2012-05-30

**Authors:** A Karbasfrushan, K Farhadi, J Amini-Saman, S Bazargan-Hejazi, A Ahmadi

**Affiliations:** 1 Department of Anesthesiology, Critical Care and Pain Management, Imam Reza Hospital, Kermanshah University of Medical Sciences, Kermanshah, Iran; 2 College of Medicine, Charls Drew University of Medicine and Sciences, Los Angeles, CA, USA; 3 Semel Institute for Neuroscience and Human Behavior, David Geffen School of Medicine, University of California, CA, USA; 4 Karolinska Institute, Stockholm, Sweden

**Keywords:** Intrathecal, Bupivacaine, Midazolam, Cesarean Section, Pain

## Abstract

**Background:**

The benzodiazepines are used primarily for anxiolysis, amnesia and sedation. However, recent investigations have shown that some forms of this group of drugs have also direct effect on pain. This study aims to determine the effect of midazolam in reducing the severity of pain in women scheduled for elective cesarean section.

**Methods:**

In a prospective, double blind randomized controlled trial, two groups parallel study, was conducted in Imam Reza/Moatazedi Hospital, an affiliate of Kermanshah University of Medical Sciences. Parturient women who met study inclusion criteria were consecutively assigned into either experimental (n=62) or control groups (n=62). Women in the experimental group received bupivacaine (10 mg) plus intrathecal midazolam (2 mg/ml) (BM) and those in the control group received bupivacaine plus normal saline (BNS). The outcome pain severity was measured by Verbal Numerical Rating Scale.

**Results:**

In comparison with the BNS group, mothers in the BM group reported a significant relief in pain (15 min and 120 min) after the surgery. There were no significant differences between the groups regarding the intensity of pain 5, 30, 60 and 240 min after the surgery. The average time until the first dose of additional analgesic, per mother’s request was 142.18±55.19 min in the BNS vs 178.06±77.33 min in the BM group.

**Conclusion:**

Combination of bupivacaine plus intrathecal midazolam was an effective anesthetic technique to provide improvement in pain. The onset of sedation was faster in the BM group compared with the BNS group. The duration of effective analgesia, and the time for regression of sensory analgesia was the same in both groups in our study. However, incidence of nausea and vomiting was higher in the experimental group.

## Introduction

The benzodiazepines are used primarily for anxiolysis, amnesia and sedation.[1] However, recent investigations have shown that some forms of this group of drugs have also direct effect on pain. Some investigators have suggested that spinal cord benzodiazepine receptors can play an important role in producing sufficient analgesia, further clarifying the underlying mechanism of action in this class of drugs.[2][3][4][5][6] It has further been argued that intrathecal midazolam reduces excitatory GABA-mediated neurotransmission in interneurons, leading to a decrease in the excitability of spinal dorsal horn neurons.[6] In animal studies, researchers have been able to show that intrathecal midazolam by binding to benzodiazepine receptors in the spinal cord increases the threshold for pain.[7][8][9][10] In a more recent study, Sen and colleagues[11] reported that in women with caesarean section delivery, intrathecal midazolam (2 mg) when added to 1.5 ml of 5% lignocaine showed relief in post operative pain. Similar result was provided by Tucker and colleagues.[12] Other investigators have also shown that intrathecal-administered midazolam when added to bupivacaine significantly improved the duration and quality of spinal anesthesia;[2] produced a more effective and longer analgesia in perianal and lower-extremity surgeries;[13][14] prolonged the duration of spinal blockade in orthopedic patients,[15] and provided postoperative early recovery of motor function in orthopedic and diabetic mellitus patients undergoing foot debridement.[16][17] In majority of these studies, no serious adverse effects were reported.

The long-term administration of intrathecal midazolam infused with clonidine has also provided promising evidences of almost immediate and complete pain relief in four patients with refractory chronic benign pain. Even with continuous use, these patients did not show any sign of building tolerance against the drug, no toxic effect on nerve roots were reported and other side-effects were minimal.[18]

As indicated in the aforementioned studies, while intrathecal midazolam has been used for the management of different types of pains in different type of patients, but its analgesic effect in women undergoing cesarean section is less documented. Twenty years after the introduction of intrathecal use of midazolam, providing relief from pain after cesarean section delivery with fewer side effects remains challenging for anesthesiologists.[19]

This study was an attempt to investigate the analgesic effect of intrathecal midazolam using a double blind randomized study of 124 pregnant women scheduled for elective cesarean section. The objective of this study was to determine the effect of midazolam in reducing the severity of pain in women scheduled with cesarean section. The underlying hypothesis was that intrathecal injection of midazolam interrupted the spinal cord pathways taken by pain afferents.

## Materials and Methods

This was a prospective, randomized double blind, two groups parallel study conducted in Imam Reza/Moatazedi Hospital, an affiliate of Kermanshah University of Medical Sciences. Pregnant women, 18-45 years of age with ASA I and II who were scheduled for elective cesarean section were screened for the study eligibility criteria.

Prior to screening, the potential participants for eligibility, study coordinator explained details of the research protocol and gave them an ample amount of time to ask questions from the coordinator and/or physicians who were involved in the trial. Subsequently, eligible parturients signed the informed consent. The principle of the study protocol was approved by the Ethic Committee of Kermanshah University of Medical Sciences.

Parturient women were included in the study if they met the following criteria: 1) Age between 18-45 years, 2) Had no severe spinal lesion, 3) Had no sign of abscess or infection of skin and soft tissue in the location of needle insertion, 4) Did not report severe anxiety and restlessness, 5) Had no sign of lesion in the central nervous system; 6) Did not present peripheral nerve lesion and 7) Did not screen positive for presence of significant mental disorders or drug abuse, and agreed to sign a written informed consent. Study sample, therefore, included 124 consented pregnant women aged 18-45 years who met all the study inclusion criteria.

Parturient women were consecutively assigned into either experimental (n=62) or control groups (n=62). Both the investigators and the subjects were blinded to the randomization, using numbered unlabled 5 ml syringe containing study’s analgesic medication. Before induction of epidural anesthesia, all parturients received an infusion of lactated ringer’s solution (10 ml/kg). Standard monitoring devices including electrocardiogram, finger tip pulse oximeter, and non invasive blood pressure (NIBP) were used to measure the hemodynamic variables. Women in the experimental group received bupivacaine (12.5 mg/2.5 ml) plus intrathecal preservative-free midazolam (2.0 mg/ml) (BM) and those in the control group received bupivacaine (12.5 mg/2.5 ml) plus normal saline (1 ml) (BNS). Midazolam, at a concentration of 5 mg/ml formulations was diluted in 0.9% sodium chloride till the solution reached 2 mg/ml. Subarachnoid injection was performed, while the parturient was in the right lateral decubitus position. Injection was administered with a 24-gague Quincke spinal disposable needle at the L3-4 or L4-5 intervertebral space. Immediately after the subarachnoid injection, parturient was turned supine and her head was rested on a pillow. The time to successful onset and the levels of anesthesia were determined and recorded. Surgery was performed when sensory block at or above T6 was determined. Oxygen was administered routinely by face mask at 6 liter min-1 until the end of the operation. The time of the surgery, time of delivery and termination of the surgery were recorded as well.

Vital sign parameters included blood pressure, pulse rate, respiratory rate, and pulse oximetry that were recorded at 5, 10, 20, 30, 45, 60, 120, and 240 min. Incidence and frequency of complications including nausea, vomiting, shivering, and itching, as well as their clinical managements that were recorded and monitored intra-operatively and postoperatively. Time to regression of sensation by 2 dermatomes as well as time patient reported complete recovery from anesthesia and mother’s motor function was assessed and registered postoperatively. Mothers’ level of sedation/alertness was subjectively assessed using the Observer Assessment of Alertness/Sedation (OAA/S) by the study anesthesiologist who rated mother’s responsiveness to verbal stimuli (i.e. repeating her own name) on a scale of 1=unresponsive to 5=alert. This was also recorded at 5, 15, 30, 60, 120, and 240 min, postoperatively.

Severity of pain was measured by Verbal Numerical Rating Scale (VNRS). Mothers were asked to rate their pain from a scale of 0=no pain to 10=worse pain possible. Pain scores were recorded at 5, 15, 30, 60, 120, and 240 min, postoperatively. The newborn condition was assessed by the APGAR scores in the 1st and 5th minute of delivery.

The study data is presented as mean±SD. Independent t-test (for quantitative data) and Mann-Whitney U test (for qualitative data) were used to determine differences between the experimental and control group means. The level of significance was established at p<0.05. All the statistical analyses were performed using SPSS software (version 15, SPSS Inc, Chicago, IL, USA).

## Results

One hundred and twenty four women ages 18 to 45 years (mean=32 years) who were scheduled for elective cesarean section were enrolled. There were no significant differences between the two groups prior to the surgery with regard to mean age, height, weight, and gestational age. Anesthesia was achieved in 5.36±1.908 min in the control group and in 3.64±1.856 min in the experimental group and the difference between the two groups was significant (p=<0.001). The duration of analgesia in control group (BNS) was 107.77±43.273 vs 115.71±61.36 min in the experimental group (BM) (p>0.05).

In compare with the BNS group, mothers in the BM group reported better relief in pain (15 min, (p=0.006 and 120 min, p=0.007) after the surgery. Although, there were no statistically significant differences between the groups regarding the intensity of pain 5, 30, 60, 240 min after the surgery (Table 1). The average time until the first dose of additional analgesic, per mother’s request, was 142.18±55.19 min in the BNS vs 178.06±77.33 min in the BM group (p=< 0.021).

**Table 1:** Postoperative comparison of pain intensity in the study groups.

	**Pain** **5-min**	**Pain** **15-min**	**Pain** **30-min**	**Pain** **60-min**	**Pain** **120-min**	**Pain** **240-min**
**Mean±SD** **BM Group **	0.48	0.45^a^	0.79	108	2^a^	3.24
**Mean±SD** **BNS **	0.76	1.21^a^	1.61	3.15	4.79^a^	7.03

^a^ (P<0.05), bupivacaine midazolam: BM; bupivacaine normal saline: BNS

Table 2 shows that there was a significant difference between BM group when compared to BNS group regarding the report of nausea (p=0.006). The incidence of nausea and vomiting was higher in the BM group. However, there were no significant differences between the BM group and BNS group in respect to mean HR, systolic blood pressure, itching, trembling, and need for extra or emergency treatment 5, 10, 20, 30, 45, 60, 120, and 240 min, post-surgery. No significant differences were detected between the two groups in regard to their level of awareness 5, 15, 30, 60, 120, and 240 min after the injection (p>0.05). The APGAR score of the newborns at 1st and 5th min of delivery in both groups were similar and newborns were all in good condition.

**Table 2:** Postoperative comparison of nausea and vomiting in the study groups.

	**BM^a^ Group** **F (%)**	**BNS^b^ Group** **F (%)**	**Total**
**Nausea and vomiting**			
**• Yes**	33 (53.2)	18 (29.0)	51 (41.1)
**• No**	29 (46.8)	44 (71.0)	73 (58.9)
**Total**	62 (100)	62 (100)	124 (100)

^a^ Bupivacaine midazolam: BM

^b^ Bupivacaine normal saline: BNS

## Discussion

This study showed that intrathecal midazolam produced a highly significant postoperative pain relief for the experimental (BM) 15 and 120 min groups. This finding was supported by previous studies. Tucker et al.[12] reported that among women in labor combination of intrathecal midazolam and fentanyl provided significant pain relief and did not increase incident of any maternal adverse event or abnormalities on the cardiotocography. Comparable result was reported by Shah and colleagues who conducted a prospective, randomized, and observer blinded study that involved 60 patients (30/group) undergoing minor and intermediate lower abdominal surgery. They concluded that the quality of postoperative pain relief improved in the experimental group who received buprenorphine and bupivacaine supplemented with intrathecal midazolam (2 mg).[20] Similarly, in a double blind study conducted by Kim,[21] intrathecal midazolam significantly increased the analgesic effect of spinal blockade among patients undergoing haemorrhoidectomy. Others also observed that intrathecal midazolam produced postoperative pain relief for patients with chronic lumbar pain and for women undergoing caesarean section delivery while also having antiemetic effect.[22][23] However, among children undergoing inguinal herniorrhaphy, combination of bupivacaine and midazolam did not provide any further analgesic advantages in comparison to children who received bupivacaine alone.[24]

Findings from human studies also pointed to the analgesic effect of intrathecal midazolam. In one study, intrathecal administration of midazolam (0.3-2 mg dissolved in 3 ml of 5% dextrose) in nine patients with acute pain reduced post-operative somatic pains but not visceral pain.[25] In a prospective, double blind, randomized–controlled study, Serrao and colleagues[26] compared the therapeutic effects of epidural methyl prednisolone (80 mg) with intrathecal midazolam (2 mg) among 28 patients with chronic low back pain. They concluded that patients in both groups reported similar improvement in activity and sleep related pain as well as sensory and affective components of their pain experience two months post treatment. However, every patient who was treated with steroid reported taking either the same or more analgesic medication after the treatment, whereas one-third and half of the midazolam-treated patients reported that they took less medication during the same two months follow-up period. Intrathecal therapy using midazolam also have been more effective to reduce neuropathic pain in cancer patients,[27][28] and when added to bupivacaine increased analgesia and shortened the recovery time among diabetic mellitus patients undergoing foot debridement.[5] Valentine et al.,[29] further reported that intrathecal midazolam had detectable analgestic effect post-operatively in women with elective caesarean section.

The mechanism that leads to antinoception effect of midazolam has been the subject of debates among various investigators. Findings from animal studies (i.e., adult rats) suggested that midazolam exerted its effect at the neural level by reducing excitatory synaptic transmission i.e., acting on the gamma aminobutyric acid type A/benzodiazepine receptor in interneurons, leading to a decrease in the excitability of spinal dorsal horn neurons.[6][30][31] In an earlier study, Edwards et al.[32] concluded that the antinoception action of intrathecal midazolam was due to the mediating role of benzodiazepine-GABA receptor complex within the spinal cord which enhanced GABA activity in the primary afferent neurons.

Our study showed that intrathecal administration of midazolam did not affect mother’s heart rate, blood pressure, or incidence of hypotension, itching, trembling, and need for extra emergency treatment. Also the condition of the newborns, evaluated by AGPAR score, was good in both groups. Similar results have been reported by others.[11][12][19]

On note, however, it that the incidence of nausea and vomiting, that are the most common side effects of intrathecal analgesia, were more frequent among mothers in the BM group (53.2%) than mothers in the BNS group (29.0%). While Valentin and others did not detect such side effects attributable to midazolam.[29][33] Further studies are needed to determine the dose-response relationship between intrathecal midazolam and incidence of nausea and vomiting among women undergoing elective caesarean section.

It was further observed in our study that the onset of sedation was 1.72±0.052 faster in BM group than in BNS group. Similar results have been reported by previous studies. Sajedi and Islami [4] stated that administration of 5 mg midazolam shortened the onset of sensory block and time to peak effect. Kim and colleagues also concluded that time to first analgesia was significantly greater in the midazolam groups than the placebo group.[21] As for the duration of effective analgesia, it was the same in both the BM and BSM groups, which supports previous findings.[34][35] However, in Sajedi and Islami’s study, the durations of motor and sensory blocks were significantly longer in the group with 5 mg dose of midazolam in comparison to the other two groups in the study. Data from other studies confirms their findings. In the Shah’s study, the duration of postoperative pain relief was longer in the experimental group. In a study by Prakash et.al., the mean duration of postoperative analgesia among women undergoing cesarean section was moderately longer in the group that received 2 mg dose of intrathecal midazolam.[36]

While some histological studies in animals have provided empirical evidence against neurotoxicity of midazolam,[8][37][38] yet other investigators have warned against its use in human, and have suggested needs for further studies.[39][40][41][42] Rigler and colleagues,[43] on the other hands, suggested that clinicians should consider using lower concentration of the drug and adopt a “ceiling” dose for local anesthetic to avoid possible neurotoxicity. In a cohort study investigating the neurological damage of intrathecal midazolam, Tucker et al.[44] reported that among the cohort with intrathecal midazolam (2 mg), a one week and one month post operative follow-up showed no sign of increased risk of neurologic symptoms in comparison to those who received intrathecal anesthesia without midazolam. In the current study, we used intrathecal midazolam (2 mg), but further study with appropriate follow up is needed to replicate Tuker’s results.

Our findings are limited since we were not able to evaluate the dose-response relationship of intrathecal midazolam. Further studies should be conducted to evaluate different doses of intrathecal midazolam on pain response among women undergoing cesarean section. Also, appropriate follow-up with the mothers are need to evaluate symptoms or adverse effects that may persist beyond expected recovery period.

The present study demonstrated that combination of bupivacaine (10 mg) plus intrathecal midazolam (2 mg) was an effective anesthetic technique to provide improvement in pain. The onset of sedation was faster in the group that received bupivacaine plus intrathecal midazolam compared with those who received bupivacaine plus normal saline. The duration of effective analgesia and the time for regression of sensory analgesia was the same in both groups in our study. However, incidence of nausea and vomiting was higher in the experimental group.
